# Somatic and germline aberrations in homologous recombination repair genes in Chinese prostate cancer patients

**DOI:** 10.3389/fonc.2023.1086517

**Published:** 2023-03-29

**Authors:** Yixiao Liu, Bo Jin, Cheng Shen, Xianshu Gao, Xin Qi, Mingwei Ma, Hongzhen Li, Han Hao, Qi Tang, Kaiwei Yang, Yue Mi, Jie Guan, Xuero Feng, Zhisong He, Haixia Li, Wei Yu

**Affiliations:** ^1^ Department of Urology, Peking University First Hospital, Peking University, Beijing, China; ^2^ Institute of Urology, Peking University, Beijing, China; ^3^ National Urological Cancer Center, Beijing, China; ^4^ Department of Clinical Laboratory, Peking University First Hospital, Beijing, China; ^5^ Department of Radiation Therapy, Peking University First Hospital, Beijing, China; ^6^ Department of Geriatrics, Peking University First Hospital, Beijing, China

**Keywords:** prostate cancer, DNA repair, mutation, China, genetic testing

## Abstract

**Simple summary:**

Somatic and germline aberrations in homologous recombinant repair (HHR) genes are associated with increased incidence and poor prognosis for prostate cancer. Through next-generation sequencing of prostate cancer patients across all clinical states from north China, here the authors identified a somatic mutational rate of 3% and a germline mutational rate of 3.9% for HRR genes using 200 tumor tissues and 714 blood specimens. Thus, mutational rates in HRR genes were lower compared with previous studies.

**Background:**

Homologous recombination repair deficiency is associated with higher risk and poorer prognosis for prostate cancer. However, the landscapes of somatic and germline mutations in these genes remain poorly defined in Chinese patients, especially for those with localized disease and those from north part of China. In this study, we explore the genomic profiles of these patients.

**Methods:**

We performed next-generation sequencing with 200 tumor tissues and 714 blood samples from prostate cancer patients at Peking University First Hospital, using a 32 gene panel including 19 homologous recombination repair genes.

**Results:**

TP53, PTEN, KRAS were the most common somatic aberrations; BRCA2, NBN, ATM were the most common germline aberrations. In terms of HRR genes, 3% (6/200) patients harbored somatic aberrations, and 3.8% (28/714) patients harbored germline aberrations. 98.0% (196/200) somatic-tested and 72.7% (519/714) germline tested patients underwent prostatectomy, of which 28.6% and 42.0% had Gleason scores ≥8 respectively. Gleason scores at either biopsy or prostatectomy were predictive for somatic aberrations in general and in TP53; while age of onset <60 years old, PSA at diagnosis, and Gleason scores at biopsy were clinical factors associated with positive germline aberrations in BRCA2/ATM.

**Conclusions:**

Our results showed a distinct genomic profile in homologous recombination repair genes for patients with prostate cancer across all clinical states from north China. Clinicians may consider to expand the prostate cancer patients receiving genetic tests to include more individuals due to the weak guiding role by the clinical factors currently available.

## 1 Introduction

Prostate cancer (PCa) is the second most common cancer in men worldwide (1). Despite PCa incidence in China remains far lower than that in western countries, it has increased rapidly in recent years due to lifestyle changes and screenings (2). Genomic and molecular complexity in prostate cancer have been tremendously profiled (3–7). Although many correlations between different genetic variants and PCa clinical outcomes are yet to be revealed, patients with pathogenic variants in DNA damage repair genes, especially those from homologous recombinant repair (HRR) such as ATM, BRCA2, and BRCA1, are found to be closely associated with younger onset, increased risks, and poorer prognosis (8, 9). Furthermore, platin based regimens and poly (ADP-ribose) polymerase inhibitors have been proved to have additional benefits in this specific subgroup (10, 11).

However, most of these findings came from studies enriched with non-Asian population, and research data on domestic Chinese patients are still inadequate. Previous studies have found unique genomic and epigenomic features in Chinese PCa patients, implying the importance of taking population-specific contexts into consideration (12). There are a series of studies addressing the HRR genes in Chinese population (13–19). These studies have provided outlines of mutation landscape in HRR genes in Chinese PCa patients. However, most of these studies came from south part of China. Furthermore, these studies focused mostly on germline variants and metastatic cases. Integrative analysis of somatic variants somatic and germline alterations in Chinese PCa patients is still limited.

In this study, we sequenced 714 fresh blood samples and 200 formalin-fixed paraffin-embedded (FFPE) PCa tissues from a total of 720 PCa patients across all disease states at Peking University First Hospital (PUFH). The majority of patients in this PUFH cohort came from north China. We also analysed clinical characteristics and obtained the surgical results of these patients. Thus, PUFH cohort would serve as a unique supplementation to the current bank of HRR gene alterations in Chinese population.

## 2 Materials and methods

### 2.1 Patients

From September 2019 to February 2022, a total of 721 consecutive patients treated at Peking University First Hospital were enrolled in this study with histologically confirmed prostate adenocarcinoma. Among them, 194 patients underwent paired somatic and germline sequencing; 6 patients underwent somatic sequencing solely; 520 patients underwent germline sequencing solely. Thus, a total of 200 patients underwent somatic sequencing, and a total of 714 patients underwent germline sequencing. 196 out of the 200 somatic tested patients and 519 out of the 714 germline tested patients also underwent radical prostatectomy (RP). For the 196 patients underwent RP, somatic sequencing was carried out using RP specimens; for the 4 patients who did not undergo RP, somatic sequencing was done using biopsy specimens. For both types of specimens, pathologists checked under microscope to make sure the proportion of the tumor region accounted for >20% of total area. Peripheral blood drawn from patients was used for germline sequencing. Clinical data were collected from the institutional medical database. The study was approved by the Committee for Ethics at Peking University First Hospital.

### 2.2 Sample preparation, sequencing and variant classification

DNA samples were sequenced with a HRR 32-gene panel (including 19 HRR genes: ATM, ATR, BRCA1, BRCA2, BARD1, CHEK1, CHEK2, FANCA, FANCL, PALB2, RAD51B, RAD51C, RAD51D, RAD54L, CDK12, NBN, PPP2R2A, BRIP1, MRE11A, and 13 therapeutic related genes: AR, BRAF, CDH1, ESR1, HDAC2, KRAS, TP53, NRAS, PIK3CA, HOXB13, ERBB2, PTEN, STK11) provided by Amoy Diagnostics, Xiamen, China. DNA was extracted and sheared into 200 to 500 bp fragments and then used for library construction using the HRR gene combination detection library preparation kit (Amoy Diagnostics, Xiamen, China) according to the manufacturer’s protocol. DNA sequencing was performed on the NextSeq500 Illumina platform (Illumina, San Diego, CA, USA) at an average depth of 1000×.

The types of detected mutations included single nucleotide variants (SNVs) and small indels (<50 bp). Sequencing data was analyzed by Sequencing Data Analysis Software (Amoy Diagnostics, Xiamen, China). In terms of the quality control for calling, a sample would not pass the quality control if: the total depth of mutation and wild-type alleles was lower than 100× for somatic sequencing or 20× for germline sequencing; the depth of a mutation allele was lower than 5×; the allelic frequency was lower than 3% for somatic sequencing or 20% for germline sequencing; the base quality of a mutation was lower than 30; the base quality of mutation allele minus the average base quality of both mutation and wild-type alleles was smaller than -2; the read quality of mutation allele minus the average read quality of both mutation and wild-type alleles was smaller than -2; the mapping quality of mutation allele minus the average mapping quality of both mutation and wild-type alleles was smaller than -0.3. These parameters had been validated in previous studies (20, 21).

For annotation, we followed the evidence framework recommended by American College of Medical Genetics and Genomics (ACMG) guidelines (22). In terms of population data, we first searched for the presence and frequency of each variant in 1000G, ExAc, gnomAD (23–25). Then, for disease databases, we searched in ClinVar for each variant’s pathogenicity (26), and BRCA 1/2 variants were also searched in BRCA Exchange (27). Somatic variants were also searched in Cancer Hotspots, OncoKB, and JAX-CKB (28–30). The assessment also included searching the scientific and medical literature. Next, computational predictive programs were used to predict the function of each variant: Mutation Taster, PolyPhen-2, PROVEAN, and SIFT for non-synonymous mutations (31–34); HSF, NNsplice for synonymous mutations (35, 36). In this study, germline mutations included pathogenic (P) and likely pathogenic (LP) variants.

### 2.3 Statistical analysis

We used Mann-Whitney U test for clinical characteristics between different subgroups, including age at diagnosis, Gleason score (GS), et al. All reported P values were 2-tailed, and α <.05 was considered statistically significant. SPSS Statistics 25 was used for data analysis, and OriginPro 2020b and ComplexHeatmap package in R 4.2.1 were used for drawing figures.

We further compared the mutation frequency in this cohort with other public or published data. For somatic variants, we analysed PRAD patients from The Cancer Genome Atlas (TCGA) database, SU2C/PCF cohort, MSK-IMPACT cohort, and PCa patients from Chinese Prostate Cancer Genome and Epigenome Atlas (CPGEA) (4, 6, 12). For germline variants, we analysed results from studies by Nicolosi et al. which enriched with western PCa patients and Zhu et al. which enriched with south China PCa patients, as well as SU2C/PCF and MSK-IMPACT cohorts (4, 5, 7, 15). For SU2C/PCF and MSK-IMPACT cohorts, the data were accessed through cBioportal (37, 38).

## 3 Results

### 3.1 Patient characteristics

The characteristics of the 200 patients underwent somatic testing and 714 patients underwent germline testing were listed in **Tables 1**, **2** respectively. The majority of the patients came from north part of China. Patients mostly came from Beijing and Hebei, followed by Shandong, Liaoning, Inner Mongolia, and Shanxi. Most patients were Han Chinese, although patients who were not Han were also recruited. For somatic testing, 21% of the participants were below 60-year-old. 66.5% belonged to high to very high-risk groups, while 7.5% metastasised to regional lymph nodes and 2.5% to distant organs. For germline testing, 19.9% were younger than 60-year-old at time of diagnosis. 57.6% belonged to high to very high-risk groups according to NCCN clinical practice guideline, while 9.2% had regional lymph node involvement and 17.6% had remote metastasis.

**Table 1:** Clinical characteristics of somatic testing at PKUFH.

		Total (%)	Without PV/LPV (%)	PV/LPV (%)	HRR+ (%)	BRCA2/1/ATM+ (%)	TP53+ (%)	PTEN+ (%)
Number of patients		200 (100)	174 (87)	26 (13)	6 (3)	3 (1.5)	10 (5)	5 (2.5)
Birthplace								
North part								
Beijing		80 (40)	74 (42.5)	6 (23.1)	3 (50)	1 (33.3)	3 (30)	0 (0)
Hebei		29 (14.5)	25 (14.4)	4 (15.4)	1 (16.7)	1 (33.3)	1 (10)	1 (20)
Henan		4 (2)	3 (1.7)	1 (3.8)	0 (0)	0 (0)	0 (0)	1 (20)
Heilongjiang		6 (3)	6 (3.4)	0 (0)	0 (0)	0 (0)	0 (0)	0 (0)
Jilin		6 (3)	6 (3.4)	0 (0)	0 (0)	0 (0)	0 (0)	0 (0)
Liaoning		9 (4.5)	6 (3.4)	3 (11.5)	1 (16.7)	1 (33.3)	1 (10)	0 (0)
Inner Mongolia		8 (4)	6 (4)	2 (7.7)	0 (0)	0 (0)	0 (0)	0 (0)
Shandong		19 (9.5)	16 (9.2)	3 (11.5)	1 (16.7)	0 (0)	2 (20)	0 (0)
Shanxi		8 (4)	5 (2.9)	3 (11.5)	0 (0)	0 (0)	1 (10)	1 (20)
Shaanxi		4 (2)	3 (1.7)	1 (3.8)	0 (0)	0 (0)	0 (0)	1 (20)
South Part								
Anhui		3 (1.5)	3 (1.7)	0 (0)	0 (0)	0 (0)	0 (0)	0 (0)
Fujian		1 (0.5)	1 (0.6)	0 (0)	0 (0)	0 (0)	0 (0)	0 (0)
Guangdong		2 (1)	1 (0.6)	1 (3.8)	0 (0)	0 (0)	1 (10)	0 (0)
Guizhou		1 (0.5)	1 (0.6)	0 (0)	0 (0)	0 (0)	0 (0)	0 (0)
Hubei		3 (1.5)	2 (1.1)	1 (3.8)	0 (0)	0 (0)	1 (10)	0 (0)
Hunan		3 (1.5)	3 (1.7)	0 (0)	0 (0)	0 (0)	0 (0)	0 (0)
Jiangsu		6 (3)	5 (2.9)	1 (3.8)	0 (0)	0 (0)	0 (0)	1 (20)
Jiangxi		1 (0.5)	1 (0.6)	0 (0)	0 (0)	0 (0)	0 (0)	0 (0)
Zhejiang		5 (2.5)	5 (2.9)	0 (0)	0 (0)	0 (0)	0 (0)	0 (0)
Chongqing		2 (1)	2 (1.1)	0 (0)	0 (0)	0 (0)	0 (0)	0 (0)
Ethnicity								
Han		192 (96)	166 (95.4)	26 (100)	6 (100)	3 (100)	10 (100)	5 (100)
Other		8 (4)	8 (4.6)	0 (0)	0 (0)	0 (0)	0 (0)	0 (0)
Age of onset, y, *p value*				*0.781*	*0.214*	*0.379*	*0.485*	*0.299*
<60		42 (21)	36 (20.7)	6 (23.1)	0 (0)	0 (0)	6 (60)	2 (40)
>=60		158 (79)	138 (79.3)	20 (76.9)	6 (100)	3 (100)	4 (40)	3 (60)
PSA at diagnosis, ng/ml, *p value*				*0.104*	*0.902*	*0.108*	*0.691*	*0.370*
0-10		83 (41.5)	76 (43.7)	7 (26.9)	3 (50)	0 (0)	3 (30)	1 (20)
11-20		56 (28)	47 (27)	9 (34.6)	1 (16.7)	1 (33.3)	5 (50)	2 (40)
21-100		56 (28)	48 (27.6)	8 (30.8)	2 (33.3)	2 (66.7)	1 (10)	2 (40)
>100		5 (2.5)	3 (1.7)	2 (7.7)	0 (0)	0 (0)	1 (10)	0 (0)
GS at biopsy, *p value*				0.003*	*0.873*	*0.152*	0.027*	*0.454*
3+3		27 (13.5)	27 (15.5)	0 (0)	0 (0)	0 (0)	0 (0)	0 (0)
3+4		57 (28.5)	49 (28.1)	8 (30.8)	4 (66.7)	1 (33.3)	2 (20)	2 (40)
4+3		55 (27.5)	51 (29.3)	4 (15.4)	0 (0)	0 (0)	3 (30)	1 (20)
8		28 (14)	26 (14.9)	2 (7.7)	0 (0)	0 (0)	1 (10)	1 (20)
9-10		28 (14)	17 (9.8)	11 (42.3)	2 (33.3)	2 (66.7)	4 (40)	1 (20)
Unknown		5 (2.5)	4 (2.3)	1 (3.8)	0 (0)	0 (0)	0 (0)	0 (0)
Risk group at time of diagnosis, *p value*				0.002*	*0.447*	*0.112*	*0.099*	*0.112*
Low to intermediate		47 (23.5)	45 (25.9)	3 (11.5)	3 (50)	0 (0)	1 (10)	0 (0)
High to very high		133 (66.5)	118 (67.8)	15 (57.7)	2 (33.3)	2 (66.7)	7 (70)	4 (80)
Regional		15 (7.5)	9 (5.2)	5 (19.2)	1 (16.7)	1 (33.3)	1 (10)	1 (20)
Metastatic		5 (2.5)	2 (1.1)	3 (11.5)	0 (0)	0 (0)	1 (10)	0 (0)

*p < 0.05.

LP, likely pathogenic. P, pathogenic. HRR, homologous recombinant repair. GS, Gleason score.

Gleason score in accordance to 2014 International Society of Urological Pathology (ISUP) Consensus Conference on Gleason Grading of Prostatic Carcinoma.

Risk Group: High to very high: T3-T4 OR, Gleason score ≥ 8 OR, PSA >20 ng/mL; Regional: Any T, N1, M0; Metastatic: Any T, Any N, M1.

**Table 2:** Clinical characteristics of germline testing at PKUFH.

	Total (%)	VUS/LB/B (%)	PV/LPV (%)	HRR+ (%)	BRCA2/1/ATM+ (%)
Number of patients	714 (100)	684 (95.8)	30 (4.2)	28 (3.9)	17 (2.4)
Birthplace					
North part					
Beijing	243 (34)	234 (34.2)	9 (30)	8 (28.6)	5 (29.4)
Gansu	2 (0.3)	2 (0.3)	0 (0)	0 (0)	0 (0)
Hebei	106 (14.8)	104 (15.2)	2 (6.7)	2 (7.1)	2 (11.8)
Henan	28 (3.9)	26 (3.8)	2 (6.7)	2 (7.1)	1 (5.9)
Heilongjiang	25 (3.5)	25 (3.7)	0 (0)	0 (0)	0 (0)
Jilin	13 (1.8)	10 (1.5)	3 (10)	2 (7.1)	1 (5.9)
Liaoning	44 (6.2)	42 (6.1)	2 (6.7)	2 (7.1)	2 (11.8)
Inner Mongolia	38 (5.3)	36 (5.3)	2 (6.7)	2 (7.1)	2 (11.8)
Ningxia	3 (0.4)	2 (0.3)	1 (3.3)	1 (3.6)	1 (5.9)
Shandong	58 (8.1)	53 (7.7)	5 (16.7)	5 (17.9)	1 (5.9)
Shanxi	32 (4.5)	31 (4.5)	1 (3.3)	1 (3.6)	0 (0)
Shaanxi	11 (1.5)	11 (1.6)	0 (0)	0 (0)	0 (0)
Tianjin	7 (1)	7 (1)	0 (0)	0 (0)	0 (0)
Xinjiang	4 (0.6)	4 (0.6)	0 (0)	0 (0)	0 (0)
South part					
Anhui	16 (2.2)	15 (2.2)	1 (3.3)	1 (3.6)	1 (5.9)
Fujian	3 (0.4)	3 (0.4)	0 (0)	0 (0)	0 (0)
Guangdong	3 (0.4)	3 (0.4)	0 (0)	0 (0)	0 (0)
Guangxi	3 (0.4)	3 (0.4)	0 (0)	0 (0)	0 (0)
Guizhou	2 (0.3)	2 (0.3)	0 (0)	0 (0)	0 (0)
Hubei	6 (0.8)	6 (0.9)	0 (0)	0 (0)	0 (0)
Hunan	9 (1.3)	8 (1.2)	1 (3.3)	1 (3.6)	0 (0)
Jiangsu	20 (2.8)	19 (2.8)	1 (3.3)	1 (3.6)	1 (5.9)
Jiangxi	6 (0.8)	6 (0.9)	0 (0)	0 (0)	0 (0)
Shanghai	6 (0.8)	6 (0.9)	0 (0)	0 (0)	0 (0)
Sichuan	8 (1.1)	8 (1.2)	0 (0)	0 (0)	0 (0)
Yunnan	3 (0.4)	3 (0.4)	0 (0)	0 (0)	0 (0)
Zhejiang	11 (1.5)	11 (1.6)	0 (0)	0 (0)	0 (0)
Chongqing	4 (0.6)	4 (0.6)	0 (0)	0 (0)	0 (0)
Ethnicity					
Han	697 (97.6)	668 (97.7)	29 (96.7)	28 (100)	17 (100)
Other	17 (2.4)	16 (2.3)	1 (3.3)	0 (0)	0 (0)
Age of onset, y, *p value*			*0.620*	*0.487*	0.030*
<60	142 (19.9)	135 (19.7)	7 (23)	7 (25)	7 (41.2)
>=60	572 (80.1)	549 (80.3)	23 (77)	21 (75)	10 (58.8)
PSA at diagnosis, ng/ml, *p value*			*0.642*	*0.574*	0.038*
0-10	214 (30)	206 (30)	8 (26.7)	7 (25)	1 (6)
11-20	171 (23.9)	164 (24)	7 (23.3)	7 (25)	6 (35.3)
21-100	243 (34)	231 (33.8)	12 (40)	11 (39.3)	7 (41.2)
>100	67 (9.4)	64 (9.4)	3 (10)	3 (10.7)	3 (17.6)
Unknown	19 (2.7)	19 (2.8)	0 (0)	0 (0)	0 (0)
GS at biopsy, *p value*			*0.329*	*0.208*	0.041*
3+3	69 (9.7)	66 (9.6)	3 (10)	2 (7.1)	0 (0)
3+4	142 (19.9)	137 (20)	5 (16.7)	5 (17.9)	2 (11.8)
4+3	121 (16.9)	117 (17.1)	4 (13.3)	4 (14.3)	3 (17.6)
8	103 (14.4)	99 (14.5)	4 (13.3)	3 (10.7)	2 (11.8)
9-10	225 (31.5)	212 (3)	13 (43.3)	13 (46.4)	9 (52.9)
Unknown	54 (7.6)	53 (7.7)	1 (3.3)	1 (3.6)	1 (5.9)
Risk group at time of diagnosis, *p value*			*0.612*	*0.379*	*0.065*
Low to intermediate	105 (14.7)	98 (14.3)	7 (23.3)	6 (21.4)	1 (5.9)
High to very high	411 (57.6)	400 (58.5)	11 (36.7)	10 (35.7)	8 (47.1)
Regional	66 (9.2)	62 (9.1)	4 (13.3)	4 (14.3)	3 (17.6)
Metastatic	126 (17.6)	118 (17.3)	8 (26.7)	8 (28.6)	5 (29.4)
Unknown	6 (0.8)	6 (0.9)	0 (0)	0 (0)	0 (0)

*p < 0.05.

VUS, variants of uncertain significance; LB, likely benign; B, benign. LP, likely pathogenic; P, pathogenic. All in accordance to American College of Medical Genetics and Genomics (ACMG) laboratory guideline. HRR, homologous recombinant repair. GS, Gleason score.

Gleason score in accordance to 2014 International Society of Urological Pathology (ISUP) Consensus Conference on Gleason Grading of Prostatic Carcinoma.

Risk Group: High to very high: T3-T4 OR, Gleason score ≥ 8 OR, PSA >20 ng/mL; Regional: Any T, N1, M0; Metastatic: Any T, Any N, M1.

### 3.2 The landscape and comparative analysis of genomic aberrations

**Figure 1:**
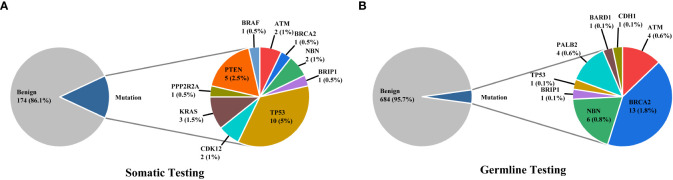
The distributions of somatic **(A)** and germline **(B)** mutations in our cohort.

**Figure 2:**
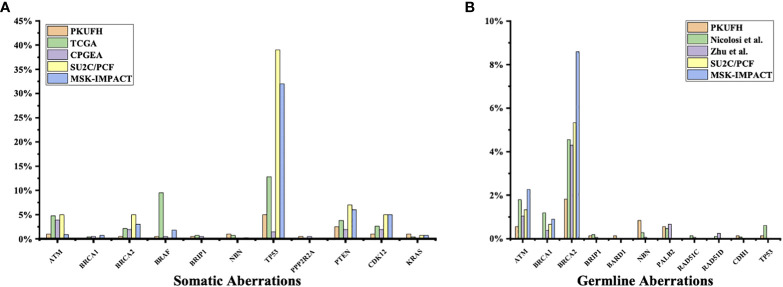
Comparison of somatic and germline variants between this study and previous ones **(A)** Somatic pathogenic variants in our study (Peking University First Hospital, PKUFH), PRAD from TCGA, Chinese Prostate Cancer Genome and Epigenome Atlas (CPGEA), SU2C/PCF, and MSK-IMPACT. CPGEA is a cohort with 208 pairs of tumor tissue samples and matched healthy control tissue from Chinese patients with primary prostate cancer. TCGA, SU2C/PCF, and MSK-IMPACT focused mostly on Caucasian populations. SU2C/PCF profiled only patients with metastatic castration resistant prostate cancer. **(B)** Germline Pathogenic variants (PV) and likely pathogenic variants (LPV) in PKUFH, studies by Nicolosi et al. and Zhu et al., SU2C/PCF, and MSK-IMPACT. The study by Nicolosi et al. recruited mostly patients with Caucasian descendance. The study by Zhu et al. recruited Chinese patients, mostly with metastatic disease.

**Figure 3:**
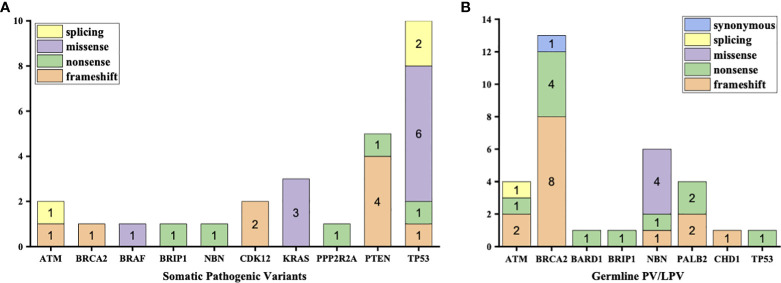
Types of mutations in each type of aberrations **(A)** Aberrations in somatic testing **(B)** Pathogenic variants (PV) and likely pathogenic variants (LPV) in germline testing.

3% individuals harbored HHR somatic mutations. 1% demonstrated mutations in ATM and 0.5% in BRCA2 (**Figure 1A**). TP53 mutations (5%) were the most commonly identified alterations, followed by PTEN (2.5%), KRAS (1.5%), ATM (1%), NBN (1%), and CDK12 (1%). The mutational rates of these genes were lower than those previously reported (**Figure 2A**). For TP53, the most common type of mutations was missense; for PTEN, the most common type of mutations was frameshift (**Figure 3A**). GS at biopsy associated significantly with somatic aberrations in general (p=0.003) and in TP53 (p=0.027) (**Table 1**). VUS frequency was 53% for somatic sequencing (Supporting File).

3.9% individuals harbored HHR germline mutations, with 1.8% for BRCA2 and 0.6% for ATM (**Figure 1B**). These were lower compared with previous studies (**Figure 2B**). 2 mutations were not in HRR pathway: one for CDH1 and one for TP53. Most common types of mutations for ATM and BRCA2 were both frameshift (**Figure 3B**). For all clinical factors, age of onset (p=0.03), PSA at diagnosis (p=0.038), and GS at biopsy (p=0.041) were significantly associated with higher risk of germline mutations in BRCA2 and ATM in PCa (**Table 2**). VUS frequency was 50.3% for germline sequencing (Supporting File).

Particularly, no aberration was identified for BRCA1 in either germline or somatic tested patient.

### 3.3 Relation of needle biopsy to radical prostatectomy Gleason score

**Tables 3**, **4** detailed the corresponding RP GS for each of the five needle biopsy GS groups. For both, approximately 80% of cases were upgraded from a needle biopsy GS 6 to higher grade at RP. Approximately 50% of the cases had matching GS 3 + 4 and 4 + 3 at biopsy and RP. For the rest of the cases in these two groups, most cases upgraded after RP. When the biopsy was GS 8, there are almost 50% of the cases upgraded to GS 9-10 after RP. A biopsy of GS 9-10 led to nearly 70% equal grading at RP. Of note, somatic aberrations in general (p=0.006) and for TP53 (p=0.013) once again were significantly associated with higher RP grades, as for GS at biopsy mentioned above.

**Table 3:** Somatic tested patients underwent radical prostatectomy (n=196).

a. Radical Prostatectomy (RP) Grades Stratified by Different Somatic Variants.						
RP Grades	Without PV/LPV	PV/LPV	HRR+	BRCA2/1/ATM+	TP53+	PTEN+
*p value*		0.006*	0.028*	0.029*	0.013*	*0.777*
**3+3**	4	0	0	0	0	0
**3+4**	55	4	2	0	1	1
**4+3**	60	5	0	0	1	3
**8**	9	3	0	1	1	0
**9-10**	34	10	2	2	5	0
**Unknown**	11	1	0	0	0	1
**b. RP Grades Stratified by Biopsy Gleason Scores**						
Biopsy GS	3+3	3+4	4+3	8	9-10	Unknown
**RP GS**						
**3+3**	4 (14.3)	0 (0)	0 (0)	0 (0)	0 (0)	0 (0)
**3+4**	12 (42.9)	33 (57.9)	9 (16.1)	2 (7.4)	2 (8)	1 (33.3)
**4+3**	7 (25)	18 (31.6)	32 (57.1)	5 (18.5)	2 (8)	1 (33.3)
**8**	0 (0)	1 (1.8)	4 (7.1)	4 (14.8)	2 (8)	1 (33.3)
**9-10**	1 (3.6)	5 (8.8)	10 (17.9)	11 (40.7)	17 (68)	0 (0)
**Unknown**	4 (14.3)	0 (0)	1 (1.8)	5 (18.5)	2 (8)	0 (0)
**Tota**l	28 (100)	57 (100)	56 (100)	27 (100)	25 (100)	3 (100)

*p < 0.05.

Unknown RP grade is due to neoadjuvant reasons.

**Table 4:** Germline tested patients underwent radical prostatectomy (n=519).

a. Radical Prostatectomy (RP) Grades Stratified by Different Germline Variants						
RP Grades	VUS/LB/B	PV/LPV	HRR+	BRCA2/1/ATM+		
*p value*		*0.971*	*0.837*	*0.307*		
**3+3**	11	1	1	0		
**3+4**	126	7	6	3		
**4+3**	123	2	2	0		
**8**	43	1	1	1		
**9-10**	168	6	5	5		
**Unknown**	29	2	2	1		
**b. RP Grades Stratified by Biopsy Gleason Scores**						
Biopsy GS (%)	3+3	3+4	4+3	8	9-10	Unknown
**RP GS**						
**3+3**	10 (15.2)	1 (0.7)	0 (0)	0 (0)	0 (0)	1 (2.7)
**3+4**	33 (50)	65 (47.8)	24 (21.8)	4 (5.8)	6 (5.9)	1 (2.7)
**4+3**	13 (19.7)	40 (29.4)	50 (45.5)	13 (18.8)	7 (6.9)	2 (5.4)
**8**	2 (3.0)	5 (3.7)	9 (8.2)	10 (14.5)	9 (8.9)	9 (24.3)
**9-10**	3 (4.6)	21 (15.4)	24 (21.8)	34 (49.3)	71 (70.3)	21 (56.8)
**Unknown**	5 (7.6)	4 (2.9)	3 (2.7)	8 (11.6)	8 (7.9)	3 (8.1)
**Total**	66 (100)	136 (100)	110 (100)	69 (100)	101 (100)	37 (100)

## 4 Discussion

**Figure 4:**
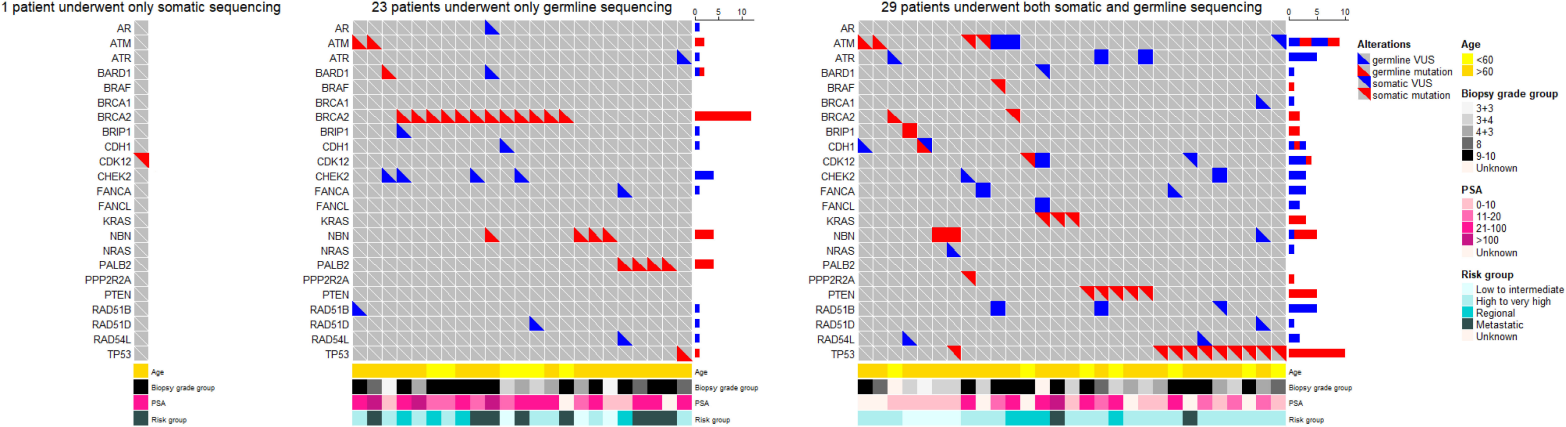
The oncoprints of somatic and germline aberrations tested using the 32-gene panel in our study. A total of 53 patients harbored either somatic or germline mutation. Among these patients, 1 patient underwent somatic sequencing only; 23 patients underwent germline sequencing only; and 29 patients underwent both somatic and germline sequencing. The VUS captured in these patients were also illustrated to provide a thorough view of genetic changes in these patients.

Through somatic sequencing of 200 cases and germline sequencing of 714 cases, we identified 53 patients harboring either somatic or germline mutations (**Figure 4**). Unlike previous studies in Chinese population, we profiled PCa in a range of clinical states—from locoregional to metastatic, and, thus, enabled comparison of genomic landscape across disease states using a single cohort. Furthermore, our study enriched PCa patients from north China, which shared a distinct genomic background but unequally represented before. Similar to previous studies, our results showed ATM and BRCA2 germline mutations were of great importance in Chinese population as were in western population. In addition, we echoed previous findings that TP53 accounted for the most common somatic mutation across all population, followed by PTEN and ATM.

The somatic mutational rates of HRR genes in this study was 3% (6/200). By contrast, 8.68% of the cases in TCGA PARD and 9.4% in MSK-IMPACT harbored somatic aberrations in HRR genes (6). All the three studies included PCa from locoregional to metastatic states. The lower somatic mutational rates in our PUFH cohort confirmed the observation that mutational rates were generally lower in Asian patients compared to Caucasian patients. However, this 3% mutational rate was also lower than 7.89% in the CPGEA cohort which only included Chinese patients (15). This might be due to the fact that our study included a smaller proportion of metastatic (2.5% VS 8.65%) and GS 9-10 (14% VS 28.8%) patients. Furthermore, 17.8% patients in SU2C/PCF harbored somatic HRR gene mutations (4). As SU2C/PCF only included individuals at metastatic castration resistant state, the lower mutational rates in our cohort confirmed that aberrations in HRR genes were less common in early state diseases.

When it came to germline alterations, 3.9% (28/714) patients carried HRR gene mutations in PUFH cohort. The germline mutational rate was 1.8% for BRCA2 and 0.6% for ATM. This was lower compared to the results reported by Nicolosi et al., SU2C/PCF, and MSK-IMPACT in Caucasian population and by Zhu et al. (4.3% for BRCA2 and 1.04% for ATM) in south Chinese population (4, 5, 7, 15). Of note, in the study by Zhu et al, 6% PCa patients had reginal lymph node metastasis and 65% had remote metastasis. However, only 9.2% and 17.6% patients had reginal lymph node and remote metastasis in our cohort. Therefore, it was once again proved that the size of the dataset, stage of disease, and patient diversity—even for the same ethnicity from different regions—were of great importance when interpreting prostate cancer genomic data.

Due to the low mutational rates for HRR genes in patients from north China, a smarter protocol to select potential candidates for genetic testing is necessary. Broadly speaking, as HRR gene mutations were associated with poor outcomes, any clinical feature potentially linked to aggressive disease could serve as a candidate marker. Currently, most guidelines addressed the use of clinical characteristics including age of onset, family history, and personal cancer history to enhance the positive rate of genetic testing (39). Nicolosi et al. argued clinical factors frequently used to identify patients who qualified germline testing, including age, race, and family history, did not correlate with positive test results in their data (7). So was GS. However, their data revealed a higher likelihood of germline mutations in HRR genes with higher stages of diseases. These findings were not echoed by those of Wu et al. In their study, Wu et al. did not observe a relationship between the presence of germline HRR gene mutations and any clinical characteristics except age at diagnosis (14). In our study, Gleason grades at diagnostic biopsy were found to be correlated with somatic aberrations in general and in TP53, while age of onset, PSA at diagnosis, and GS at biopsy were factors correlated with germline ATM and BRCA2 mutations. Due to these conflicting evidences, clinicians should be cautious using these clinical factors when prescribing tailored genetic tests for PCa patients. Indeed, it would be a better idea to expand genetic tests to include wider range of men diagnosed with PCa as much as possible due to the fact that these mutation carriers demanded radical treatment as early as possible.

As a needle biopsy and corresponding RP might not always get the same GS, we also examined the results of RP GS. Our results showed a higher incidence for GS upgrading for most groups except GS 9-10 when compared to the benchmark from Epstein et al (40). Thus, it reflected a potential bias in using biopsy GS alone to pick patients with aggressive patients. However, some pathological features including Intraductal/ductal histology and lymphovascular invasion appeared to be associated with pathogenic germline DNA-repair gene mutations in men with prostate cancer (41). Therefore, it would be reasonable for future studies to investigate the underlying morphology in these specimens possessing HRR gene mutations, and, thus, could provide extra help to smarter screening.

There were puzzles waiting to be answered. We found no BRCA1 mutation in our cohort. Interestingly, in the only one previous study focused on north China population but with a smaller size, researchers found no BRCA1 mutation neither (19). This could possibly be a distinctive genomic feature for north China individuals. Future studies should validate this observation with multiple center studies and more powerful epidemiology tools.

Our study had some limitations. First, we treated VUS as negative alterations in this study. However, preliminary yet increasing data implies the pathogenicity of some of the current VUS. Second, our panel only tested variants including single-nucleotide polymorphisms and indels, but not copy number variants, which also could result in loss of function of proteins. Third, we interpreted the somatic variants with a variety of certified knowledge bases. Of note, there was currently no ‘gold standard’ somatic mutation database that was similar to what existed for germline. These concerns warranted future efforts and advancement in clinical genetics.

In summary, we investigated the genomic landscape of both somatic and germline HRR gene alterations in the PCa patients across all clinical states from north Chinese population. The lower HRR gene mutational rate in this PUFH cohort underlined the need for a more efficient pre-testing candidate-selection protocol in genetic testing.

## Data availability statement

The datasets presented in this study can be found in online repositories. The names of the repository/repositories and accession number(s) can be found in the article/**Supplementary Material**.

## Ethics statement

The study was conducted in accordance with the Declaration of Helsinki. The studies involving human participants were reviewed and approved by Committee for Ethics at Peking University First Hospital (protocol code: 2022-199-001; date of approval: 2022-04-22). Written informed consent for participation was not required for this study in accordance with the national legislation and the institutional requirements.

## Author contributions

HXL and WY set up the experimental design and supervised the work. YL and BJ collected and selected the data. CS, XG, XQ, MM, HZL, HH, QT, KY, YM, and ZH organized the database. BJ and JG carried out the genetic testing. YL and XF performed the statistical analysis and wrote the manuscript. All authors reviewed the manuscript and agree with its contents. All authors contributed to the article and approved the submitted version. 
